# Air pollution a hidden scourge to cardiovascular health: insight from top polluted cities of Pakistan

**DOI:** 10.1097/JS9.0000000000000135

**Published:** 2023-03-03

**Authors:** Syeda T. Rehan, Aliha Iftikhar, Sanya Khan, Hassan ul Hussain, Irfan Ullah, Ushna Naseer, Muhammad Sohaib Asghar

**Affiliations:** a Dow University of Health Sciences; b Dow University of Health Sciences – Ojha Campus; c Karachi Medical and Dental College; d Jinnah Sindh Medical University, Karachi; e Kabir Medical College, Gandhara University; f Institute of Public Health and Social Sciences (IPH&SS), Khyber Medical University, Peshawar, Pakistan

*Dear Editor*,

A range of disorders impacting the cardiovascular system, which includes the heart and blood vessels, are referred to as cardiovascular diseases (CVDs). This mainly includes coronary artery disease, coronary heart disease, cerebrovascular disease, peripheral artery disease, and aortic atherosclerosis^1^. According to the WHO, 17.9 million deaths were due to CVDs in 2019, representing 32% of global deaths^2^. There are nine determining modifiable risk factors of CVD, namely smoking, diabetes mellitus, hypertension, obesity, psychosocial factors, unhealthy diet, lack of exercise, alcohol consumption, and apoB/apoA1 ratio^3^. Air pollution (AP) is known to be another risk factor and is associated with premature deaths in CVD patients in primarily low- and middle-income countries^4^. Furthermore, new research indicates that prolonged exposure to AP may raise the mortality risk of CVD patients^5^,^6^.

It has been shown in a few studies that AP increases the chance of developing CVDs such as myocardial infarction, ischemic heart disease, cardiac arrhythmia, heart failure, or cardiac arrest^5^,^6^. Particulate matter measuring 2.5 µm in diameter or less (PM_2.5_) and particulate matter measuring 10 µm in diameter or less (PM_10_) concentration are used to determine AP. As per WHO 2021 Air Quality guidelines the highest recommended average annual emission level for PM_2.5_ was 5 μg/m^3^ and for PM_10_ was 15 μg/m^3^ 7. A study by Choi *et al.* 5 showed, in patients, a higher concentration of PM_10_ was associated with an increased risk of death due to CVD. Furthermore, a study was done by Pope and colleagues, showing a statistical association between PM_2.5_ and overall cardiovascular mortality. The risk of myocardial infarction, ischemic heart disease, arrhythmia, heart failure, or cardiac arrest death was increased in polluted areas^6^. Recently Pakistan ranked as the third most polluted country globally based on an annual average PM_2.5_ concentration in 2021, by the WHO guidelines. 2021 average PM_2.5_ concentration in Pakistan is 13.4 times the WHO annual air quality guidelines^7^,^8^. The US Air Quality Index declared Lahore an unhealthy city for sensitive groups to live in^8^.

According to WHO data from 2016, about 19% of all deaths in Pakistan were due to heart diseases. However, in only 3 years, a 29% increase occurred in deaths caused by CVDs^9^. This shows a dire need to take measures to mitigate AP and prevent an increase in cardiovascular patients and cardiovascular mortality in the country.

CVD patients need multifactorial interventions, especially more intensive measures to modify individual cardiovascular risk factors with diet, drugs, exercise, weight management, complete smoking cessation, and avoidance of secondhand smoke, or combinations thereof^10^. A concerted effort should be made by the government to educate healthcare providers and at-risk patients alike about the potential health hazards of elevated AP levels through awareness campaigns. Real-time air quality information should be available in local and national media for people as implemented by the United States Environmental Protection Agency which provides air quality forecasts and reports for particle pollution available in different cities^10^. This will inform people to wear masks more often in areas badly affected by AP. Furthermore, government authorities should set stringent National Air Quality standards to lower PM_2.5_ concentration and take measures to reduce such as but not limited to, restricting open burning of waste and regulations to mitigate emissions from vehicles and factories. The government should make policies to reduce the use of fossil fuels for energy and replace it with increased construction and use of solar power and hydropower plants. Pakistan needs to protect its vulnerable population from AP-associated CVDs, otherwise, the death rate through CVDs could increase from the already appalling 29% in the near future.

## HIGHLIGHTS

- Multiple studies have associated air pollution (AP) with an increased risk of CVDs and CVD-associated mortality.
- The mortality rate in Pakistan has seen a 29% increase over three years due to CVDs.
- This article calls out the Government and respective authorities to take precautionary measures as constantly striking AP may appall the CVD associated mortality in the near future.

## Ethical approval

Not applicable.

## Sources of funding

None.

## Authors’ contribution

S.T.R. and A.I. conceived the idea. S.K., U.N., H.u.H., I.U., and M.S.A. collected the data. S.T.R. and M.S.A. analyzed and interpreted the data. I.U. and S.T.R. did write up of the manuscript. M.S.A. reviewed and revised the manuscript for intellectual content critically. All authors approved the final version of the manuscript.

## Conflicts of interest disclosure

The authors declare that they have no financial conflict of interest with regard to the content of this report.

## Research registration unique identifying number (UIN)

None.

## Guarantor

Muhammad Sohaib Asghar.

## Provenance and peer review

Externally peer reviewed, not commissioned.
